# Congenital central nervous system malformations in piedmontese calves—part 2. structural spinal cord disorders

**DOI:** 10.3389/fvets.2026.1977752

**Published:** 2026-10-26

**Authors:** Sara Ferrini, Frine Scaglione, Giulia Cagnotti, Giuliano Borriello, Carlo Cantile, Çağla Aytaş, Angela Maria Catania, Giorgia Di Muro, Elena Grego, Luca Manassero, Andrea Quaglino, Carlotta Tessarolo, Laura Tomassone, Alberto Valazza, Claudio Bellino, Antonio D'Angelo

**Affiliations:** 1 Department of Veterinary Science, University of Turin, Turin, Italy; 2 Department of Veterinary Science, University of Pisa, Pisa, Italy; 3 ANABORAPI, Associazione Nazionale Allevatori Bovini Razza Piemontese, Cuneo, Italy; 4 Istituto Zooprofilattico del Piemonte, Liguria e Valle D’Aosta, Turin, Italy

**Keywords:** bovine neurology, Chari-like malformation, spina bifida, spinal aplasia, Split cord malformation

## Abstract

**Introduction:**

Structural spinal cord malformations in cattle are likely underreported, and their true incidence, clinical spectrum, and etiological factors remain poorly characterized. We characterized congenital structural brain malformations in Piedmontese calves in a companion study, whereas the present study focuses on congenital spinal cord anomalies and describes their clinical and pathological features.

**Methods:**

In this prospective observational study (2021–2025), Piedmontese calves with suspected congenital central nervous system anomalies underwent clinical and neurological examinations, cerebrospinal fluid analysis, diagnostic imaging, molecular testing for regionally relevant teratogenic viral pathogens, necropsy and histopathology. The study population originated from the provinces of Cuneo and Turin (Italy), which together account for over 80% of Piedmontese cattle farms. Only calves with necropsy-confirmed structural spinal cord anomalies were included.

**Results:**

Of the 70 calves undergoing necropsy for suspected congenital central nervous system anomalies, structural spinal cord malformations were confirmed in 5. Spinal cord anomalies included myelodysplasia (4/5), hydrosyringomyelia (3/5), segmental spinal cord aplasia (2/5), and diplomyelia (2/5). Open spina bifida was identified in 2/5 cases and closed spina bifida in 1/5. Concurrent musculoskeletal, and urogenital abnormalities were common. Molecular testing for bovine viral diarrhea, blue tongue, and Schmallenberg viruses was negative in all cases.

**Discussion:**

In this prospective study, structural spinal cord malformations identified in Piedmontese calves comprised a heterogeneous spectrum of developmental disorders frequently associated with one another and with musculoskeletal and urogenital congenital abnormalities. This is the first study to establish a phenotypic framework for future investigations into their developmental mechanisms and genetic basis in Piedmontese cattle.

## 1 Introduction

Congenital disorders of the central nervous system (CNS) in cattle are often underrecognized. Lack of structured surveillance, the low economic value of affected calves, and reliance on post-mortem confirmation mean that many cases go undetected, especially in perinatal death or when signs are non-specific (1). A recent retrospective study reported that CNS malformations accounted for about 15% of neurological cases, ranking them third among differential diagnoses after infectious and metabolic disorders (2). Congenital spinal cord malformations comprise a heterogeneous group of developmental disorders resulting from abnormalities of gastrulation, neurulation, and spinal cord development. Reported abnormalities include spina bifida associated malformations such as meningocele, myelomeningocele, and myeloschisis; type 1 and type 2 split cord malformation also referred to as diastematomyelia and diplomyelia, respectively; segmental spinal cord aplasia/hypoplasia and, hydrosyringomyelia. These malformations may occur either in isolation or in association with other congenital defects (1, 3). Although sporadically reported in cattle, their classification and clinicopathological description is largely missing. The available literature is limited to isolated case reports and small case series (1, 4–8). Consequently, the clinicopathological spectrum and etiological background of these disorders remain incompletely understood.

Similar to congenital brain malformations, spinal cord defects may arise from genetic abnormalities (5) or intrauterine exposure to teratogenic agents, including viral infections (8), nutritional deficiencies, or ingestion of toxic plants during critical stages of embryonic development (1, 3). The underlying cause remains undetermined in most naturally occurring bovine cases, however.

With the present study we wanted to characterize the clinical presentation, computed tomographic (CT) findings, gross and histopathological features of naturally occurring congenital structural spinal cord malformations in Piedmontese calves. This study complements our previous investigation of structural intracranial malformations (9) in the same population and provides a description of developmental spinal cord disorders. Using a structured approach based on active case identification, we prospectively characterized the clinical, imaging, gross pathological and histopathological features of frequently encountered spinal anomalies. By integrating ante-mortem and post-mortem findings, this study provides a clinicopathological characterization of these disorders and relevant information for diagnosis, prevention, and future investigations into their pathogenesis and possible genetic determinants.

## 2 Materials and methods

### 2.1 Study design and animal enrolment

This prospective observational study involved Piedmontese calves examined at the Veterinary Teaching Hospital of the University of Turin between February 2021 and March 2025 for suspected congenital structural anomalies of the CNS. The study was carried out in collaboration with ANABORAPI and the voluntary participation of farmers and farm veterinarians from the provinces of Cuneo and Turin, where over 80% of Piedmontese cattle farms (est. 4,200) are located. On average, a farm maintains about 35 breeding females, with an expected annual production of roughly 115,000 calves (10). Breeders and local veterinarians were informed through an article published in Razza Piemontese, a journal familiar to Piedmontese cattle farmers, and through online meetings. Participation in the study was voluntary, and cases were included only when referred by the farm veterinarians; therefore, the study population represents a referral-based sample and may have been subject to selection bias. The study population was calves that exhibited neurological signs since birth and had either died spontaneously or were euthanized following assessment of prognosis and practical management considerations, including economic factors, as well as stillborn calves with evident skull or skeletal abnormalities. An additional inclusion criterion was the post-mortem examination documentation. Calves were excluded if neurological signs were attributed to acquired (non-congenital) conditions based on pathological findings or if pathological data were insufficient to support classification. Calves with confirmed congenital anomalies not involving the spinal cord were excluded.

### 2.2 Clinical and diagnostic evaluation

Clinical and neurological examinations of live calves were performed by a board-certified neurologist (ADA). A standardized examination protocol was applied, based on the authors’ clinical experience and the available literature on neurological examination in this species (11). Complete blood count, serum biochemistry and cerebrospinal fluid (CSF) analysis were performed in addition to diagnostic procedures, including molecular analyses for common in utero viral infections (Schmallenberg virus, SBV; blue tongue virus, BTV, and bovine viral diarrhea virus, BVDV), on either blood or spleen samples in all cases. These viruses were specifically selected as they are recognized teratogenic infectious agents associated with congenital malformations in cattle in Europe. A one-step real-time reverse transcription-polymerase chain reaction (RT-PCR) protocol was employed for their detection. The assay targeted the partial NS3 gene for BTV and the S segment for SBV (12), and the 5’-UTR for BVDV (13). CT was performed after natural death or euthanasia by sedation with xylazine hydrochloride (0.2 mg/kg intravenously, Rompun 2%, Elanco, Sèvres, France) followed with embutramide, mezebonium and tetracaine (Tanax, Intervet International B. V., Boxmeer, The Netherlands—0.1 mL/kg intravenously into the jugular vein).

CT images were acquired using a 16-slice scanner (Somatom Emotion 16, Siemens Healthineers; slice thickness 1.5 mm, 110 kV and 130 mA). Images were acquired using soft tissue and bone reconstruction kernels. Multiplanar reconstructions (MPR) and three-dimension volume rendering (3D-VR) were generated using Horos software.

### 2.3 Post-mortem examination

All calves underwent full body post-mortem examination. The entire spinal cord was examined macroscopically. and immersion-fixed in 10% neutral buffered formalin and processed for paraffin embedding. For histopathological examination, samples were collected from all areas showing gross lesions and from representative regions throughout the spinal cord, including the cervical, cervicothoracic, thoracolumbar, and lumbosacral segments (when present). Four-μm tissue sections were stained with hematoxylin and eosin (H&E), Nissl staining, and Luxol Fast Blue (LFB). Immunohistochemistry was performed on selected histological sections using various immunohistochemical cell markers. The final sample cohort consisted of cases confirmed at necropsy to have a structural spinal cord lesion.

## 3 Results

A total of 70 calves with suspected congenital CNS anomalies underwent necropsy. A total of 29/70 (41%) was excluded because necropsy revealed a non-congenital CNS disease acquired ex-utero. Twenty-five of 70 (36%) were excluded because they presented congenital abnormalities unrelated to the CNS: 6/25 (24%) had isolated microphthalmia without evidence of CNS involvement, whereas 19/25 (76%) had musculoskeletal or craniofacial malformations without associated spinal cord abnormalities, including arthrogryposis and scoliosis in 16/19 and palatoschisis in 3/19. A further 11/70 (16%) calves were not considered here because they had congenital structural brain malformations and were included in our companion study on congenital brain disorders. The remaining 5/70 (7%) had confirmed congenital spinal cord malformations and constituted the final study sample.

Of the 5 calves with congenital spinal cord malformations, 3 were presented alive and subsequently euthanized, whereas 2 were submitted as stillborn. Spinal cord anomalies included myelodysplasia (4/5), hydrosyringomyelia (3/5), segmental spinal cord aplasia (2/5), and diplomyelia (2/5). Open spina bifida was identified in 2/5 cases and closed spina bifida in 1/5. One calf with open spina bifida also had a concurrent Chiari-like malformation. Concurrent musculoskeletal and urogenital abnormalities were present (Table 1). Molecular testing (RT-PCR) for BVDV, BTV, and SBV was negative in all cases. Malformations have been sporadically observed across the entire survey area, but no major differences in farm conditions were identified among the farms.

**Table 1:** Signalment, neurological examination findings, CSF analysis, molecular testing for in utero pathogens, imaging results (CT), and necropsy findings (gross and histological) of the cases congenital structural spinal cord anomalies. CSF was collected from live animals; molecular testing for in utero infections was performed on blood samples from live calves and on the spleen tissue from post-mortem cases, with RT-PCR assay targeting BVDV, BTV, and SMBV.

Case demographics	Clinical and neurological findings; ancillary laboratory analysis; CT findings	Necropsy findings	Morphologic neurological diagnosis
Case no. 1: Male; stillborn	Marked underdevelopment of the hindquarters and pelvic limbs, body length reduction In utero pathogen testing negative Total body (TB) CT (Figure 1): Mild focal kyphosis (T11–L1); dorsal L4–L5 subluxation; severely malformed/rudimentary L6 with asymmetric left vertebral body hypoplasia; incomplete dorsal fusion of the lumbar neural arches (L1–L5, most severe at L5); near-complete sacral agenesis with absent sacroiliac articulations.	Gross: upward deviation of lumbar vertebral column, agenesis of L5-L6 vertebrae, abrupt spinal cord interruption, and remaining of filamentous structure resembling rudimentary cauda equina (Figure 5B) Histopathology: lumbar spinal cord is normal. At sacral level (S2), there is incomplete duplication of the ventral median fissure associated with severe dilation and disruption of the central canal (Figure 5C). At a more caudal level, absence of the ventral median fissure with partial fusion of the ventral funiculi (Figure 5D); remnants of the notochord (Figure 5E).	Segmental spinal aplasia; closed spina bifida; sacral myelodysplasia, diplomyelia (split cord malformation type II), and hydrosyringomyelia
Case no. 2: Female; stillborn	Body length reduction In utero pathogen testing negative TB CT (Figure 2): Severe narrowing of the C3–C4 intervertebral space; severe thoracic vertebral dysgenesis (T5–caudal thoracic spine) with failure of vertebral formation/segmentation, vertebral and costal aplasia, marked right-sided scoliosis, vertebral fusion, dorsal displacement of the caudal thoracic spine, and vertebral canal discontinuity suggestive of spinal cord interruption.	Gross: absence of vertebrae from T7 to T11, and separation of vertebral remnants (~10 cm apart); spinal cord tapering at T6, forming a fibrotic narrow band of tissue; from T13 caudally, the spinal cord is normal (Figure 6) Histopathology: abrupt termination of the spinal cord at thoracic level with terminal meningo-connective tissue and meningeal thickening. Haphazardly disposed neurons and bundles of myelinated fibers in the spinal parenchyma Other extraneural findings: congenital diaphragmatic hernia with displacement of abdominal organs into the thoracic cavity; abnormal liver position with elongated diaphragmatic ligaments, prominent biliary ducts, and focal fibrous areas; malformed spleen; small cortical kidney cyst; cystic dilation of the left uterine horn; markedly compressed thorax with caudally directed last ribs and lateral cardiac compression; lungs abnormally lobulated	Segmental spinal aplasia; focal myelodysplasia
Case no. 3: Female; 2 days old	Lumbosacral meningocele; tail agenesis; cloacal malformation; hindlimb arthrogryposis and muscle atrophy; paraplegia with absent deep pain perception CSF normal In utero pathogen testing: negative Lumbosacral CT (Figure 3): L4–L6 vertebral fusion; wedge-shaped deformity of L3–L5 with focal kyphosis; severe asymmetric hypoplasia of L6 with near-complete absence of the left dorsal portion	Gross: spina bifida with meningocele through a lumbar bony (L4-L6) and muscular defect. Histopathology: irregular disposition of neuronal bodies and myelinated fibers (Figure 7A) with moderate gliosis (Figure 7B); migration defect of spinal ganglion cells (Figure 7C); hypomyelination of the nerve roots Other extraneural findings: agenesis of the left kidney; hypoplastic uterus and urinary bladder.	Open spina bifida with meningocele; myelodysplasia
Case no. 4: Female; 2 days old	Lumbosacral meningocele (Figure 1B); tail agenesis; cloacal malformation; hindlimb arthrogryposis and muscle atrophy (Figure 1A); paraplegia with absent deep pain perception; mild proprioceptive deficit also affecting frontlimbs CSF normal In utero pathogen testing negative TB CT (Figure 3): Near-complete collapse of the T13–L1 and L2–L3 intervertebral spaces with irregular opposing endplates; mild thoracolumbar kyphosis (T13–L3); fusion of L6 with a severely hypoplastic S1 remnant; incomplete dorsal closure of the vertebral arches from L4 caudally, with complete absence of the dorsal vertebral arch at L6; near-complete sacral agenesis with absent sacroiliac articulations; complete coccygeal agenesis; severe bilateral tarsal malformations.	Gross: spina bifida with meningocele through a sacral bony (S1) and muscular defect; cervicothoracic epidural hemorrhage; craniocaudal elongation of the cerebellum with mild caudal cerebellar vermis herniation. Histopathology: haphazardly disposed neurons and bundles of myelinated fibers, associated with dilation and rupture of the central canal; meningeal fibrosis with arachnoid microcysts; multifocal hemorrhage (Figure 7D); hypomyelination of the nerve roots (Figure 7E) Other extraneural findings: hypoplasia of the sternohyoideus muscle; hypoplastic uterus and urinary bladder; rectovaginal fistula.	Chiari—like malformation complex with open spina bifida; myelodysplasia; hydrosyringomyelia
Case no. 5: Female; 5 days old	External genital malformation; hindlimb underdeveloped and atrophic; paraplegia with deep pain perception absent CSF normal In utero pathogen testing negative TB CT (Figure 4): multilevel C7–T3 vertebral fusion with combined failure of vertebral formation and segmentation; focal cervicothoracic kyphosis with moderate vertebral canal stenosis; hypoplastic thirteenth ribs (right > left); five lumbar vertebrae; partial spondylolisthesis at L2–L3 with mild vertebral canal stenosis; mild malformation of the proximal coccygeal vertebrae.	Gross: segmental enlargement of the spinal cord (T12–L2) with dilation of the central canal (Figure 8A) Histopathology: at thoraco-lumbar level, incomplete duplication of the ventral median fissure associated with severe dilation and disruption of the central canal (Figure 8B) Other extraneural findings: mild enteritis; bilateral cranial lobe pneumonia, cloacal malformation	Cervicothoracic kyphosis; diplomyelia and hydrosyringomyelia

Table 1 summarizes the clinical, CT, and pathological findings. Complete blood count, serum biochemistry, and CSF analysis were unremarkable in all cases. Supplementary Files 1–3 document the clinical manifestations observed in calves referred alive (cases nos. 3–5). The main imaging findings are presented in Figures 1–4, whereas the gross and histopathological findings are shown in Figures 5–8.

**Figure 1:**
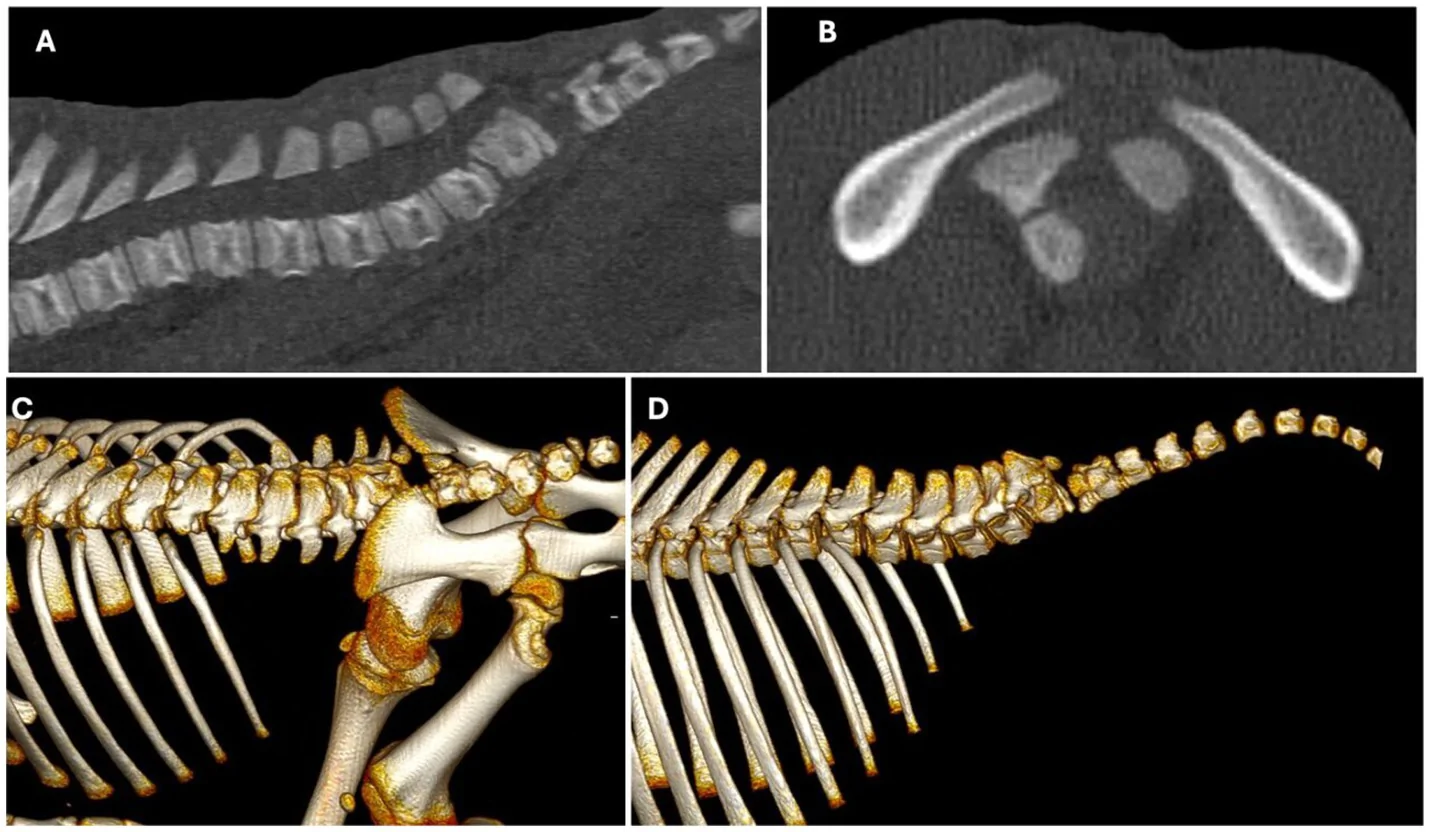
CT findings of Case no. 1. **(A)** Multiplanar reconstruction (MPR), bone window, sagittal view of the lumbosacral vertebral column showing mild focal kyphosis (T11–L1), dorsal subluxation of L4–L5, and sacral agenesis. **(B)** Transverse MPR, bone window, at the level of L6 showing a malformed vertebra with asymmetric hypoplasia of the left vertebral body. **(C)** Three-dimensional volume-rendered (3D-VR) reconstruction showing sacral agenesis with absence of the sacroiliac articulations. **(D)** 3D-VR reconstruction of the lumbosacral region following digital removal of the pelvic bones to improve visualization of the lumbosacral junction.

**Figure 2:**
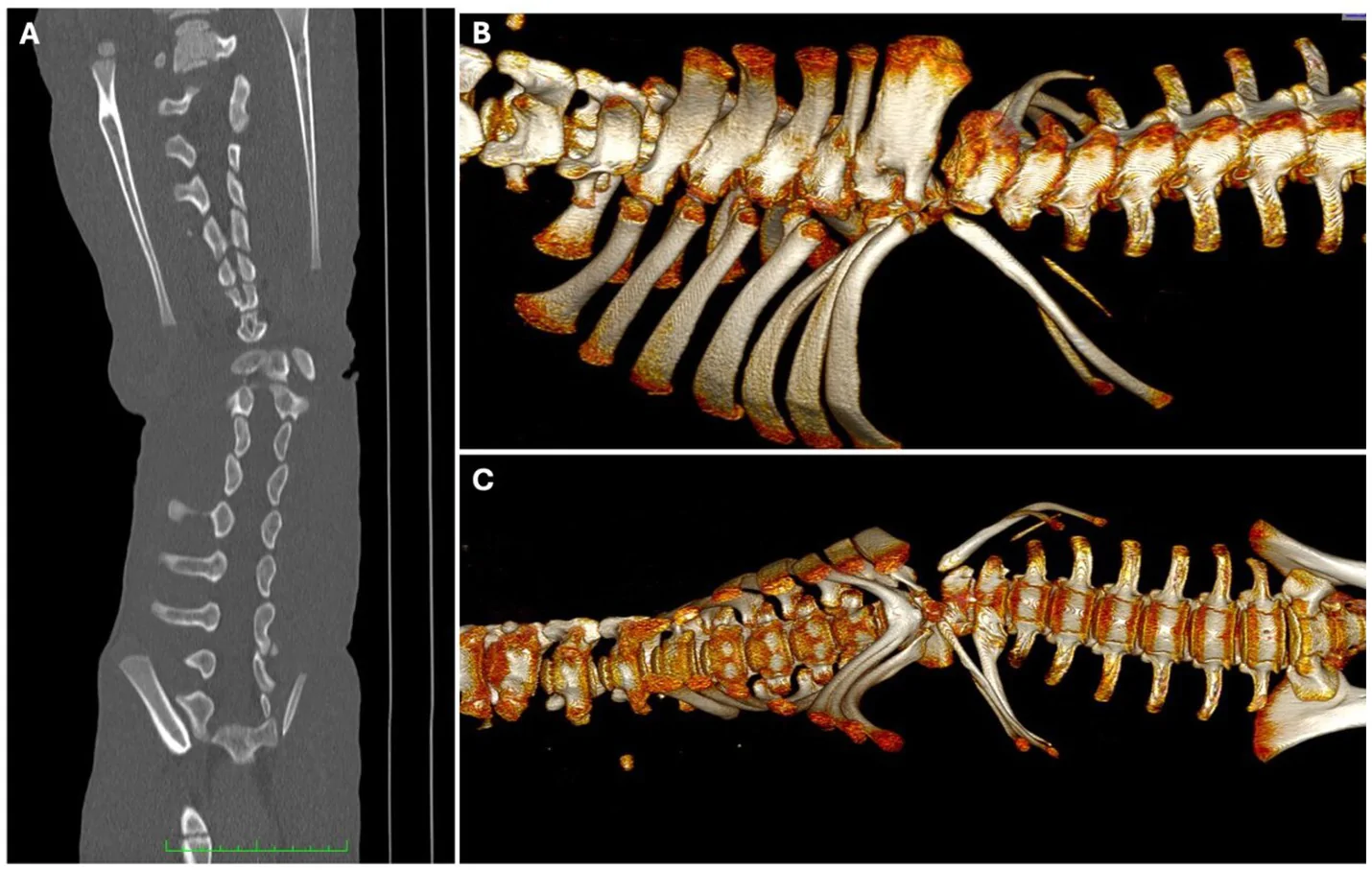
CT findings of Case no.° 2. **(A)** MPR, bone window, dorsal view showing thoracolumbar scoliosis associated with segmental vertebral agenesis. **(B)** 3D-VR reconstruction showing fusion of the vertebrae cranial to T5, including the vertebral bodies and dorsal spinous processes, with interruption of the vertebral column caudal to T5 due to segmental vertebral agenesis. **(C)** Ventral view of the 3D-VR reconstruction demonstrating fusion of the vertebrae and ribs cranial to T5, together with marked thoracolumbar scoliosis.

**Figure 3:**
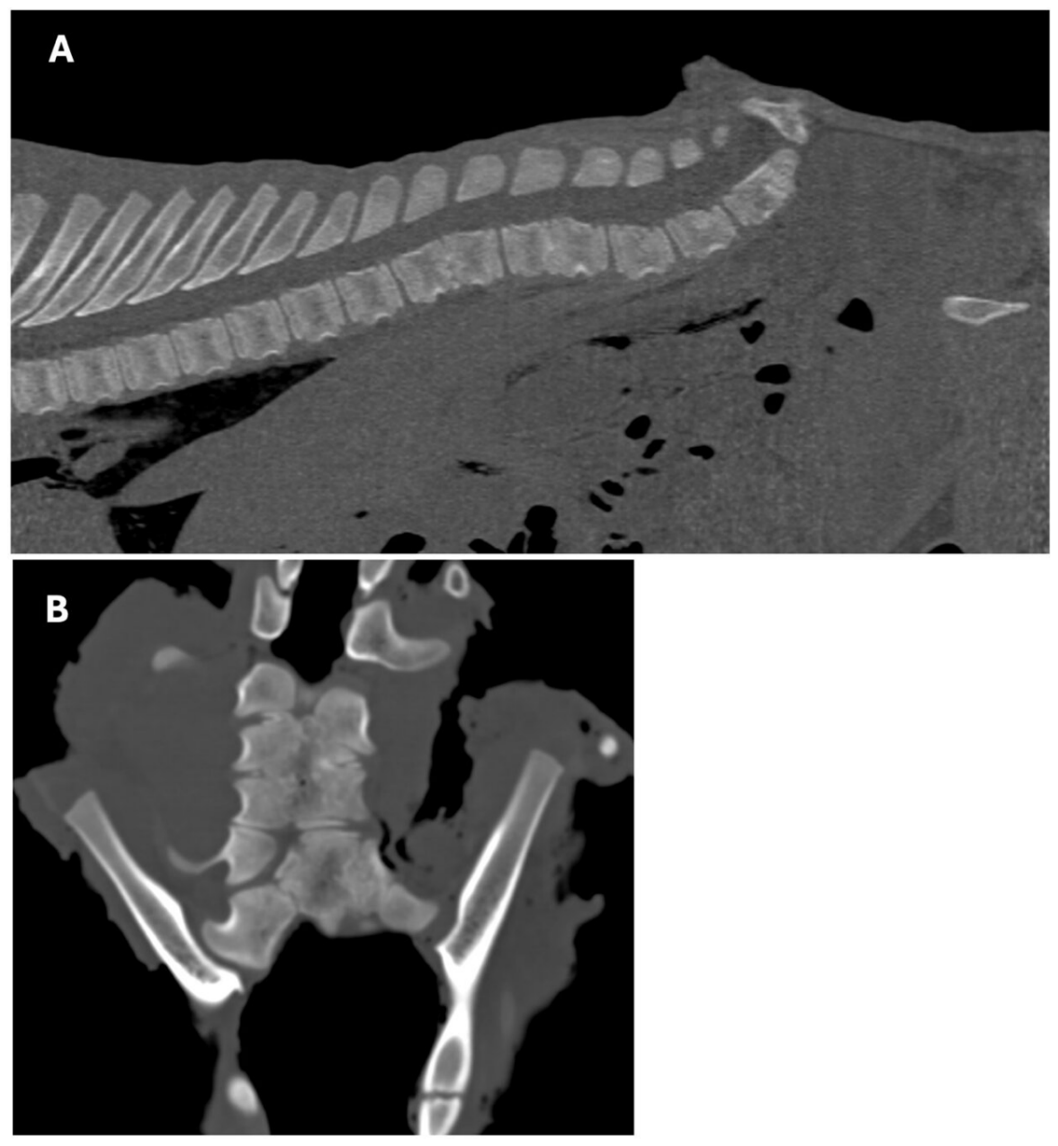
CT findings of Case nos. 3 and 4. **(A)** Case no.4. MPR, bone window, sagittal view of the lumbosacral vertebral column showing near-complete collapse of the T13–L1 and L2–L3 intervertebral spaces with irregular opposing endplates, mild thoracolumbar kyphosis (T13–L3), fusion of L6 with a severely hypoplastic S1 remnant, and incomplete lumbosacral dorsal closure of the vertebral arches. **(B)** Case no. 3. MPR, bone window, dorsal view showing rudimentary sacroiliac articular facets bilaterally.

**Figure 4:**
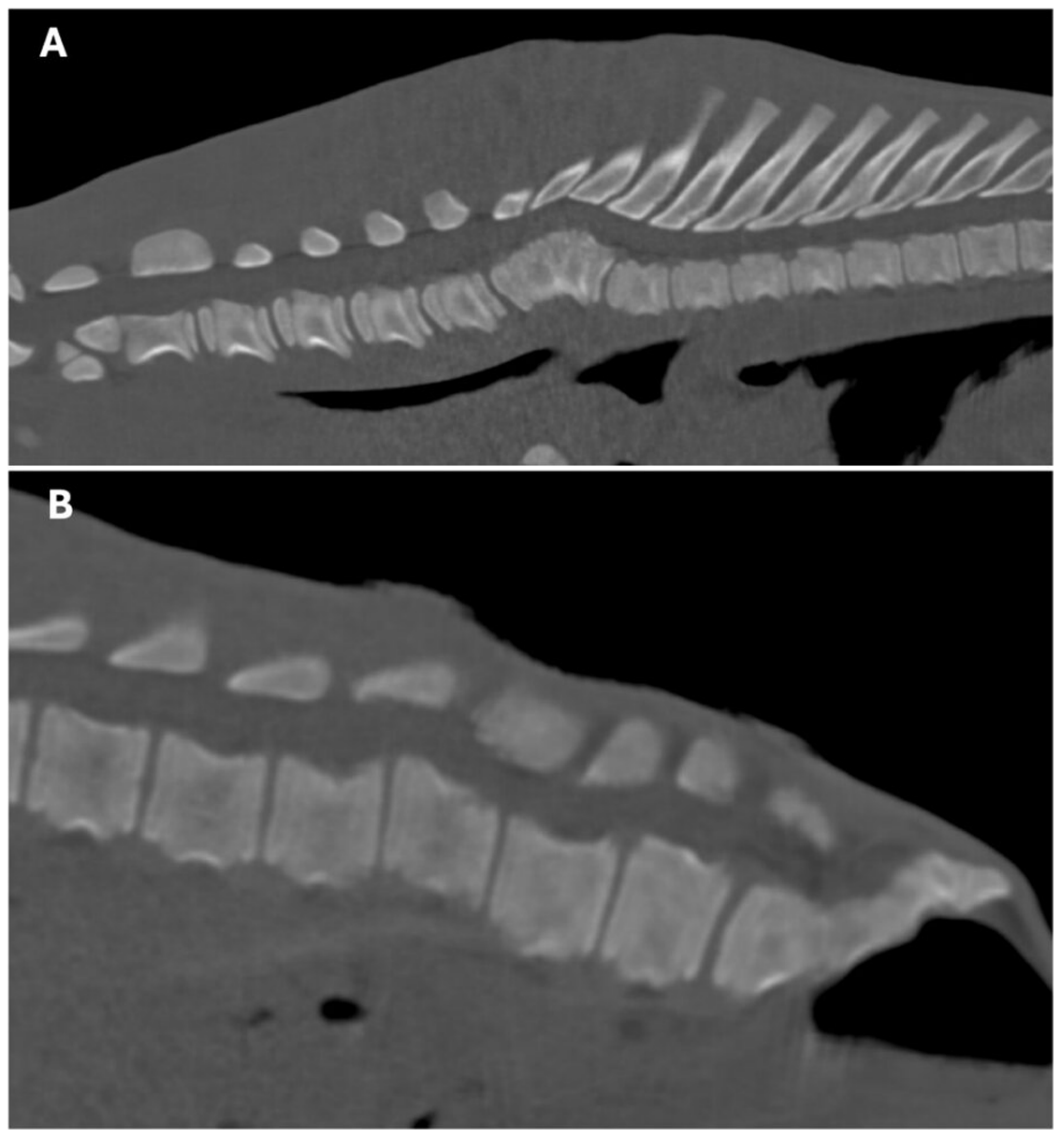
CT findings of Case no. 5. **(A)** MPR, bone window, sagittal view of the cervicothoracic vertebral column showing multilevel fusion of the C7–T3 vertebrae, associated with focal cervicothoracic kyphosis. **(B)** MPR, bone window, sagittal view of the lumbosacral vertebral column showing partial spondylolisthesis at L2–L3 and mild malformation of the proximal coccygeal vertebrae.

**Figure 5:**
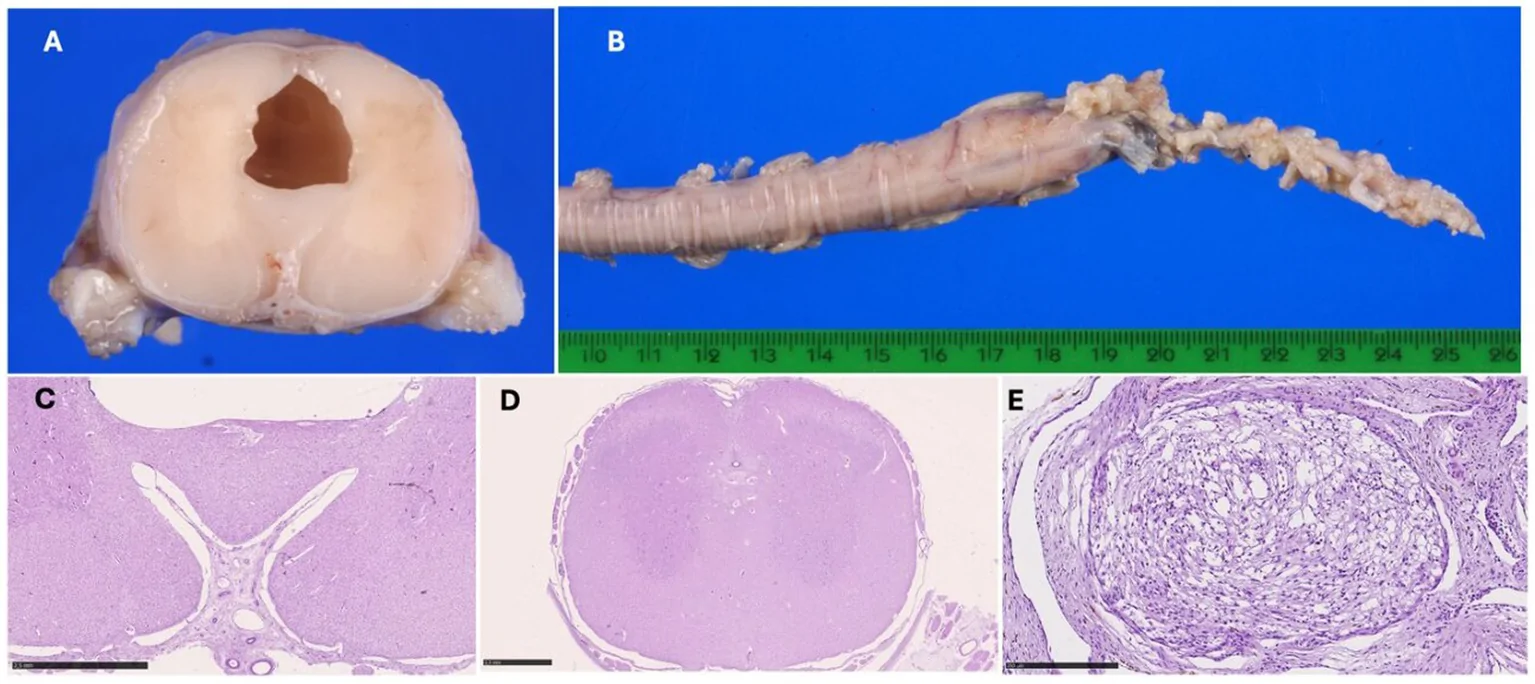
Case no. 1. Gross and histopathological findings. **(A)** Gross transverse section of the spinal cord showing marked dilation of the central canal. **(B)** Gross specimen demonstrating abrupt segmental interruption of the spinal cord. **(C)** Partial duplication of the ventral median fissure and marked dilatation of the central canal. H&E, bar = 2.5 mm. **(D)** Absence of the ventral median fissure with fusion of the ventral funiculi. H&E, bar = 2.5 mm. **(E)** Remnants of the notochord composed of physaliphorous cells. H&E, bar = 250 μm.

**Figure 6:**
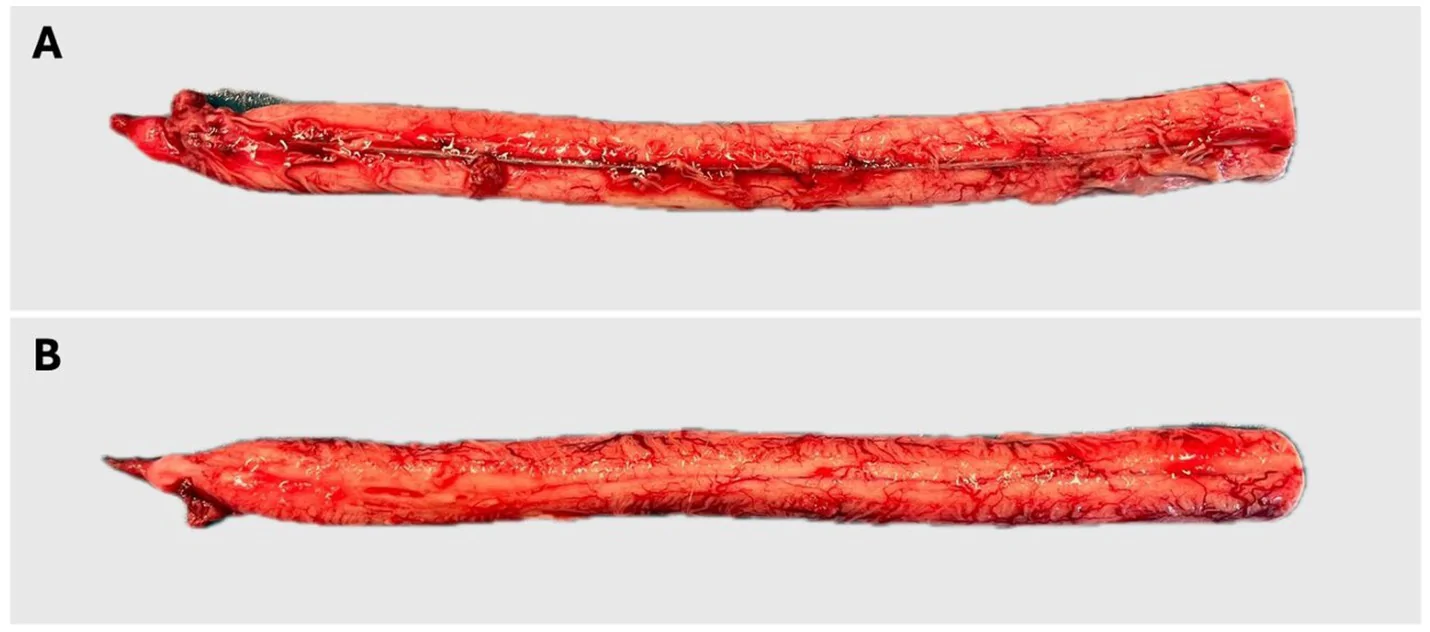
Case no. 2. Gross findings. Ventral **(A)** and dorsal **(B)** views of the thoracolumbar spinal cord (T12–L4 segment, transected at L4). Cranial is to the left and caudal to the right.

**Figure 7:**
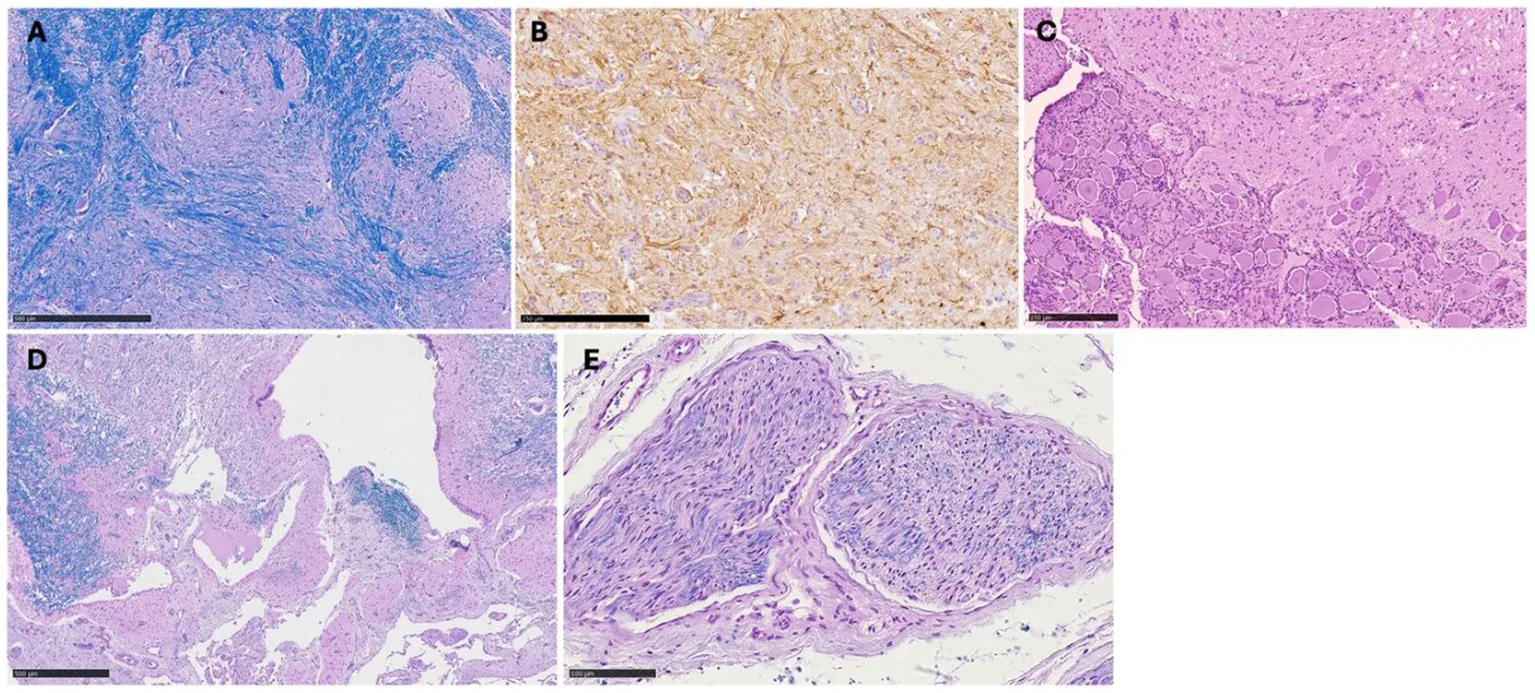
Case nos. 3 and 4. Histopathological findings. **(A)** Case no. 3. Clusters of neurons are interspersed within bundles of irregularly oriented myelinated fibers. LFB, bar = 500 μm. **(B)** Case no. 3. Gliotic tissue associated with abundant capillaries. GFAP IHC, bar = 250 μm. **(C)** Case no. 3. Aberrant disposition of spinal ganglion cells. H&E, bar = 250 μm. **(D)** Case no. 4. Dysplastic spinal cord with dilated central canal associated with tissue cavitation, arachnoid fibrosis, and hemorrhage. LFB, bar = 500 μm. **(E)** Case no. 4. Reduced myelination of the nerve roots. LFB, bar = 100 μm.

**Figure 8:**
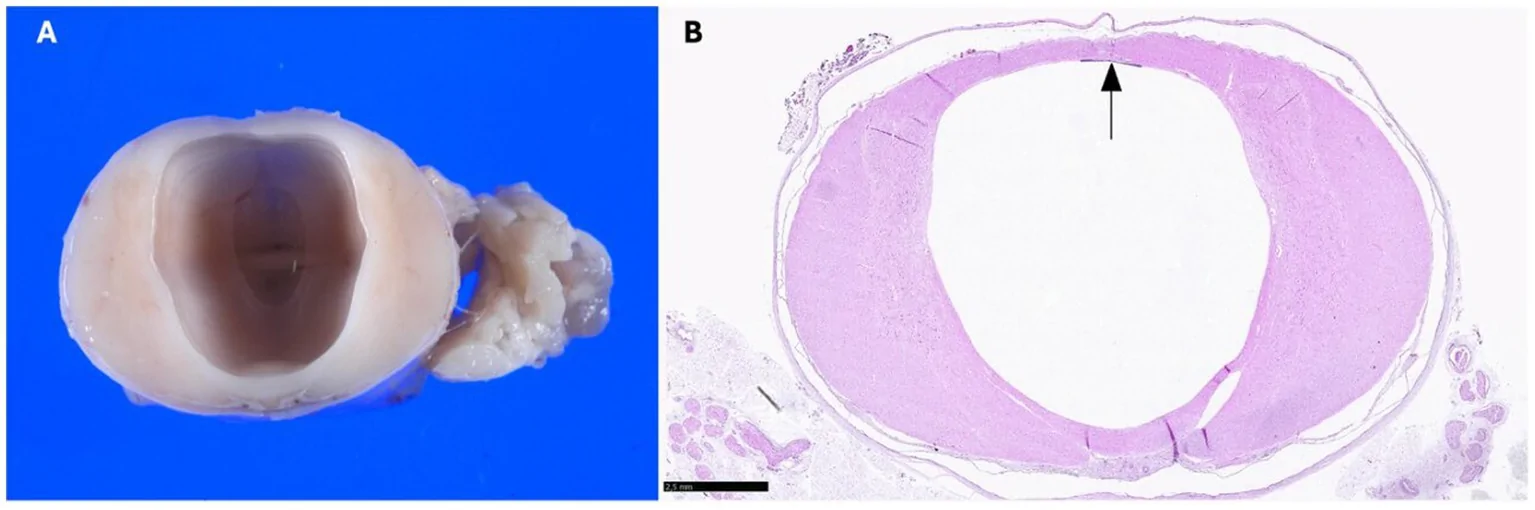
Case no. 5. Gross and histopathological findings. **(A)** Gross transverse section of the spinal cord showing marked dilation of the central canal. **(B)** Histological section. Marked dilation of the central canal with remnants of the ependymal cell layer (arrow). Note the partial duplication of the ventral median fissure. H&E, bar = 2.5 mm.

## 4 Discussion

Congenital spinal cord malformations in the examined Piedmontese calves were found to comprise a heterogeneous spectrum of spinal anomalies, including meningocele and myelodysplasia associated with spina bifida defect, type 2 split cord malformation, hydrosyringomyelia, and segmental spinal cord aplasia. Most were associated with severe vertebral malformations and congenital abnormalities affecting other organ systems, particularly the urogenital tract. Two calves were diagnosed with lumbosacral open spina bifida associated with meningocele and myelodysplasia. Both exhibited a remarkably consistent phenotype characterized by tail agenesis, cloacal malformations, urinary and genital tract abnormalities, and symmetrical hindlimb arthrogryposis. Spina bifida associated with meningocele has been described in calves (14). Although previous reports share several features with our cases, including lumbosacral localization and hindlimb arthrogryposis, the extensive caudal malformations we observed—including tail agenesis, cloacal defects, and abnormalities of the urinary and reproductive systems—have not been consistently reported.

Embryologically, spina bifida results from failure of the vertebral arches to fuse and is commonly associated with neural tube defects arising during neurulation (3). Coordinated interactions between the developing neural tube, notochord, and the surrounding mesoderm are necessary for normal formation of the vertebral column; the mesenchyme that gives rise to the axial skeleton is derived from the sclerotomal portion of the somites. These bilateral segmental structures originate from the paraxial mesoderm and are located next to the neural tube and the notochord. They begin to develop during the third week of gestation. Over the following 2 weeks, differentiation of these somites is influenced by the adjacent structures: the notochord and the neural tube stimulate the secretion of epimorphin, which induces the sclerotome cells to move close to the notochord and the neural tube and promote differentiation into vertebral cartilage and bone (3, 15).

In case no. 4, spina bifida was also associated with Chiari-like malformation. The association between spina bifida and Chiari-like malformations has been documented in cattle (5, 14, 16) and parallels the well-established association between open spina bifida and Chiari II malformation in humans (17). Although its exact pathogenesis remains unclear, Chiari malformation is thought to develop secondary to an open neural tube defect, possibly because of altered CSF dynamics during fetal development. Alterations in cranial morphology, including flattening of the calvarium, have also been implicated (5). The etiology of these malformations remains uncertain. A hereditary basis has long been suspected for bovine spina bifida, and recent studies in Danish Holstein Friesian cattle have identified several candidate genes associated with Chiari-like malformation and spina bifida (5). However, these variants were identified only in a subset of affected animals, suggesting marked genetic heterogeneity and the likely involvement of additional genetic or chromosomal abnormalities. In humans, folic acid deficiency during pregnancy is considered a risk factor, and the 5,10-methylene-tetrahydrofolate reductase (MTHFR) gene has been the most extensively investigated determinant (18). This association has not been confirmed in veterinary medicine, however, and conflicting results have been reported by studies involving dogs (19, 20). In cattle, an MTHFR polymorphism has been linked to abortion in Holsteins (21), whereas no pathogenic polymorphism was identified in a crossbreed calf with a spinal neural tube closure defect (22).

Segmental spinal cord aplasia is a rarely reported congenital malformation characterized by the focal absence or the complete interruption of a portion of the spinal cord, usually accompanied by vertebral abnormalities. Few cases have been reported in cattle, predominantly in Holstein Friesian calves (3, 23), most frequently affecting the thoracolumbar region. While MRI findings have recently been described in calves with segmental spinal cord hypoplasia, to our best knowledge, advanced imaging findings of true segmental spinal cord aplasia have not been reported. Unlike hypoplasia, which has generally been described in the absence of major vertebral abnormalities, reported cases of spinal cord aplasia are typically associated with severe vertebral malformations, particularly involving failure of vertebral arch formation.

A severe manifestation of this developmental defect has been described as perosomus elumbis, a lethal congenital syndrome characterized by agenesis of the lumbar, sacral, and coccygeal vertebrae and spinal cord, accompanied by severe musculoskeletal abnormalities of the hindquarters. Rather than constitute distinct entities, segmental spinal cord hypoplasia, segmental spinal cord aplasia, and perosomus elumbis are thought to lie along a morphological continuum of increasing severity of the same underlying developmental disturbance (24).

The embryological basis of segmental spinal cord aplasia remains uncertain. One proposed mechanism is focal disturbance of the chorda-mesoderm during gastrulation before neurulation begins. Because the chorda-mesoderm provides inductive signals for the coordinated development of the neural tube, vertebral column, and spinal nerve roots, localized disruption at a specific axial level may simultaneously result in segmental spinal cord aplasia and corresponding vertebral agenesis (24). The etiology of these rare malformations remains poorly understood. Although a hereditary basis has long been suspected, no causative mutation has yet been identified in cattle.

In the present study, split cord malformation with duplication of the ventral median fissure was identified in two calves. Both cases were also affected by hydrosyringomyelia, as previously reported in calves (7, 25). The most widely accepted embryological hypothesis for split cord malformation suggests persistence of the transient neurenteric canal, which prevents normal separation of the developing notochord from the endoderm. Consequently, the notochord splits into two hemichordal processes, resulting in bifurcation of the overlying neural plate and subsequent formation of two hemicords (26).

Finally, visceral abnormalities were noted in 4/5 calves and predominantly involved the urogenital and anorectal systems. Similar multisystem associations are well recognized in humans, in which congenital genitourinary and anorectal anomalies frequently accompany neural tube defects (27). A possible embryological explanation for this association involves disturbances of secondary neurulation. During this stage of embryogenesis, the caudal cell mass gives rise not only to the caudal spinal cord but also contributes to the formation of the vertebral column and surrounding caudal mesenchyme, which plays a central role in the development of the anorectal and genitourinary systems. Consequently, a disruption of this process may result in concurrent spinal cord malformations, tail agenesis, and defects of the urinary, genital, and anorectal tracts, as we observed (27). Of note is that 4/5 calves in the present sample were female. Although based on a small number of cases, this finding is consistent with a female predominance reported in humans and calves (22, 28).

This study has several limitations. First, despite the prospective study design and active surveillance for congenital CNS abnormalities over a 4-year period, few spinal cord malformations met the inclusion criteria. Most congenital anomalies identified during the study period involved exclusively the musculoskeletal system and were therefore excluded. The limited number of cases precludes drawing conclusions regarding the prevalence or overall phenotypic spectrum of congenital spinal cord malformations in the Piedmontese breed. The rarity and the marked heterogeneity of these malformations in cattle make large, well-characterized case series inherently challenging to collect. Nevertheless, we believe that the detailed phenotypic characterization of these cases, integrating the available clinical, imaging, pathological, and histopathological findings, provides valuable information that may support future studies in the Piedmontese breed and comparative investigations of congenital spinal cord malformations across species.

Second, although PCR testing for the main bovine teratogenic viruses yielded negative results in all cases, an infectious etiology cannot be completely excluded. The limitations of PCR testing for congenital malformations, particularly when infection may have occurred early during gestation, have been discussed in our previous study on congenital brain malformations in Piedmontese calves (9). Serological testing could provide complementary information, particularly when performed on pre-colostral serum; however, this was not available in the present study. Finally, although the absence of viral detection and other recognized nutritional or toxic risk factors raises the possibility of an underlying genetic etiology, genetic analyses were not available at the time of writing. Ongoing genomic investigations are expected to clarify the molecular basis of these malformations and determine whether the different phenotypes described here represent distinct entities or different manifestations of a common developmental disorder.

In conclusion, our study integrates clinical and CT findings, gross pathology and histopathology in the first clinicopathological characterization of congenital spinal cord malformations in Piedmontese calves. Although the precise etiopathogenesis remains to be elucidated, the absence of evidence for the main bovine teratogenic viruses and the consistent phenotypic patterns observed in several calves raises the possibility of an underlying genetic, and possibly hereditary, origin. Recognition of these conditions may facilitate clinical diagnosis, pathological classification, and breeding management. Furthermore, the detailed phenotypic characterization presented here offers a valuable framework for future genetic investigations and contributes to a better understanding of congenital spinal cord malformations in cattle, with potential comparative relevance for developmental disorders across species.

## Data Availability

The raw data supporting the conclusions of this article will be made available by the authors, without undue reservation.
